# Sting, Carry and Stock: How Corpse Availability Can Regulate De-Centralized Task Allocation in a Ponerine Ant Colony

**DOI:** 10.1371/journal.pone.0114611

**Published:** 2014-12-10

**Authors:** Thomas Schmickl, Istvan Karsai

**Affiliations:** 1 Department of Zoology, Karl-Franzens-University, Graz, Austria; 2 Department of Biological Sciences, East Tennessee State University, Johnson City, Tennessee, United States of America; Colorado State University, United States of America

## Abstract

We develop a model to produce plausible patterns of task partitioning in the ponerine ant *Ectatomma ruidum* based on the availability of living prey and prey corpses. The model is based on the organizational capabilities of a “common stomach” through which the colony utilizes the availability of a natural (food) substance as a major communication channel to regulate the income and expenditure of the very same substance. This communication channel has also a central role in regulating task partitioning of collective hunting behavior in a supply&demand-driven manner. Our model shows that task partitioning of the collective hunting behavior in *E. ruidum* can be explained by regulation due to a common stomach system. The saturation of the common stomach provides accessible information to individual ants so that they can adjust their hunting behavior accordingly by engaging in or by abandoning from stinging or transporting tasks. The common stomach is able to establish and to keep stabilized an effective mix of workforce to exploit the prey population and to transport food into the nest. This system is also able to react to external perturbations in a de-centralized homeostatic way, such as to changes in the prey density or to accumulation of food in the nest. In case of stable conditions the system develops towards an equilibrium concerning colony size and prey density. Our model shows that organization of work through a common stomach system can allow *Ectatomma ruidum* to collectively forage for food in a robust, reactive and reliable way. The model is compared to previously published models that followed a different modeling approach. Based on our model analysis we also suggest a series of experiments for which our model gives plausible predictions. These predictions are used to formulate a set of testable hypotheses that should be investigated empirically in future experimentation.

## Introduction

A social insect colony operates without a unit of central control, in consequence, individuals cannot assess pieces of global information at one specific place or from one specific nestmate. However, global colony-level information such as the current needs of the colony or the current availability and location of food [1], [2], [3] are crucial variables that social insect colonies can self-regulate as a collective [4], [5], [6]. In most cases, each worker uses only locally available information and simple rules to operate and the workers cannot compare their own knowledge to that of other nestmates. These limitations of individual workers strongly contrast with the diversity of colony level reaction to environmental changes which allow them to efficiently track environmental opportunities and challenges [7], [8], [9]. Studies on insect societies are commonly concerned with the integration of individual worker behavior into colony-level task organization and with the question of how regulation of division of labor may contribute to colony efficiency [10], [11], [12]. These societies typically develop parallel processing systems where an insect colony performs most of its operations concurrently instead of sequentially [10], [13], [14], thus frequent adjustment of the worker force engaging in different tasks is required [10], [15], [1], [16].

In the course of their evolution, ants have developed a wide range of foraging strategies [17], [3] including some very flexible and complex ones. Some of these foraging strategies can adapt to environmental changes or to the availability of the prey [18], [19], [20], what sometimes results in a flexible pattern of switching between alternative foraging strategies. For a generalist predator switching between different foraging strategies could represent an ecological advantage since it allows the exploitation of the most easily available food source from a wide range of potential preys [21], [22], [20].

**Figure 1:**
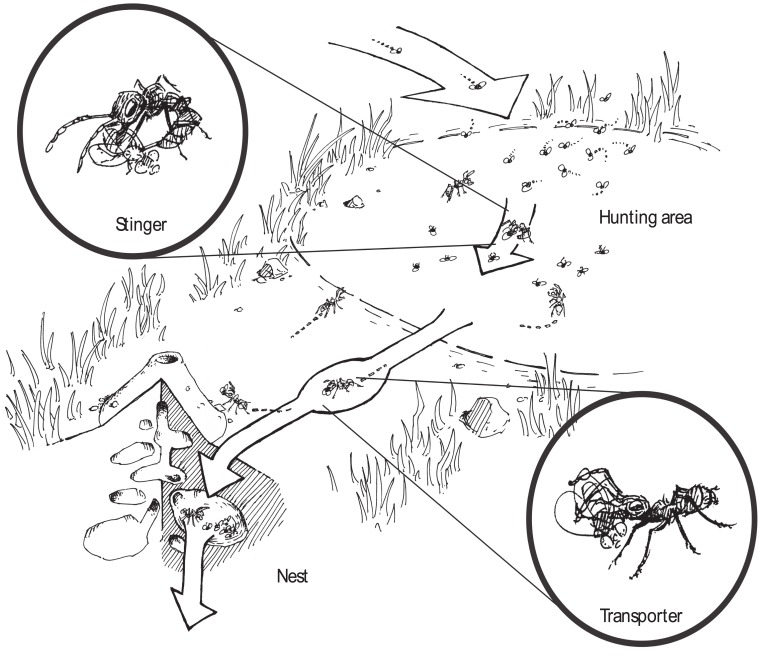
Artistic representation of the collective foraging system established by a colony of *Ectatomma ruidum*.

*Ectatomma ruidum* is a common predator in Central and Northern South America’s open forests, rainforests and plantations. It preys on a wide variety of arthropods and it also collects honey [23], [24]. In general, the foragers of this ant species hunt solitarily [25], [26], but there are two remarkable behavioral adaptations that have been observed when solitary hunting is replaced with a more complex collective strategy (Fig. 1). Schatz et al. [20] described a sophisticated graded recruitment strategy where hunting activity depends upon prey weight and size. Another strategy of adaptation in *Ectatomma ruidum* – and this is the central point of our approach – relates to the available quantity of food or prey. Schatz et al. [27] showed that in case of high density of available prey the foraging task is partitioned into 2 serialized tasks: Some ants act as “stingers”, which are specialized in killing prey and other ants act as “transporters”, which are specialized to transport corpses to the nest. A similar pattern was also found and described in *Pachychondyla caffraria* (African ponerine ant) by Agbogba and Howse [28]. Task partitioning seems to be an important form of work organization and seems to be a wide spread phenomenon in insect societies [16], [13], [29], [30], [31], [32,].

The task partitioning of *Ectatomma ruidum* shows that increased quantities of prey triggers increased recruitment of stingers in the colony. However, at certain (high) prey availability the number of stingers was found to saturate to a maximum level [27]. We reason this saturation effect as follows: If recruitment and abandonment is happening with fixed (constant) per-capita rates, then recruiting of additional workers from one task to another is a process that works slower and slower because with time many workers have already switched to the target task and less and less workers are still available in the source task. This leads to a gradual slowdown of the process that finally ends in stagnation. Comparable “saturation effects” are common in chemical reactions and in physical processes like solution of solids in fluids or vapors in gases.

The number of intermediate-inactive ants in the colony increases faster with colony size more steeply than the number of those ants engaged in hunting [33], [34]. Schatz [27] also reported that there is a significant positive correlation between the colony size and the minimal and maximal number of stingers. In addition to that, these ant colonies react more to the spatial density of prey at the hunting site than the absolute number of prey. Schatz et al. [34] also confirmed the findings of Lachaud [35] and Breed et al. [36] about the saturation of corpse delivery to the nest and conspecific thievery. In general, the stinger task group can be very effective and this could result into corpses lying around the field, waiting to be transported to the nest or to be stolen by other animals.

The specific behavior of the task groups in *Ectatomma ruidum* are described in detail by Lachaud [35]: The stinger grabs a prey animal with their mandibles and sting it until it is motionless, then the stinger drops the corpse to the ground and seeks for another prey. In some instances the transporters can solicit a corpse from a stinger if this stinger still holds its victim. However, it is more common that transporters pick up corpses from the ground and transfer these corpses to the nest. Usually, the transporter immediately returns to the hunting site for picking up another corpse after a successful delivery of a copse to the nest [34].

Schatz et al. [37] and Theraulaz et al. [38] constructed two mathematical models to describe the task partitioning of *Ectatomma ruidum* and they compared the predictions of these models to experimental data. Their models are based on the idea of simple response-threshold models [39], [15], [11], [40]. The cores of these models are nonlinear response curves. If the intensity of a stimulus, which is associated with a specific task, exceeds the response threshold of a worker, then the worker will engage in that task. In such models task partitioning emerges by the dynamic interactions of the interacting subtasks: For example performing subtask A can decrease the stimulus intensity of subtask A, but it can also increase the stimulus intensity of subtask B in parallel. This way, through well-designed threshold-curves and task interactions, self-organizing patterns of division of labor can emerge in such models.

Theraulaz et al. [38] provided experimental evidence in *Ectatomma ruidum* that the stinging and transporting behaviors are modulated by global-level stimuli such as the number of available prey and corpses respectively. Both, simulations and experiments were carried out to investigate the effect of different combinations of colony size and initial prey numbers, as these factors affect the dynamics of groups of ants which perform different tasks and the dynamics of prey and corpse quantities. These colony-level responses were followed in a two hours time scale. In the published model, the dynamics of the two worker groups (stinging and transporting), as well as the number of available prey and corpses are described by a system of four differential equations incorporating a saturation-type stimulus-response function. Schatz et al. [37] published a somewhat similar model with a sigmoid-type of stimulus-response curve resulting in strikingly similar predictions. The parameters of the model were estimated by evaluating experimental data statistically. Several predictions of the model were compared qualitatively to the data gained from the same experimental colonies. The authors concluded that their model successfully imitates what they observed in experimental colonies, after they parameterized the model from these sets of data.

In this paper we provide an alternative model of the phenomena modeled by Schatz et al. [37] and Theraulaz et al. [38]. Instead of using non-linear stimulus-response threshold functions, our main assumptions rely on regulation of different food materials by which the colony’s individual workers interact. These materials are providing information to the collective like a “common stomach”, which is an information source that is accessible for all individuals in parallel. The common stomach in our approach can be considered as a social crop, such as Karsai and Wenzel [29] described for *Metapolybia* wasps, where water is temporarily stored in the stomach of wasps. However, the term “common stomach” has a more general meaning which we adopt in this paper: A material (substance) is accumulating in a more or less well defined area and the spatial density (or “fullness of the social crop”) of this material is estimated locally through simple cues by individual workers. This local estimate then affects the workers’ behaviors, which, in turn, change the accumulation level of the material in the common stomach. This way, feedback loops emerge which provide robust regulation of material flows and task partitioning. For example, increasing the density of corpses could increase the recruitment of transporters and decrease the recruitment of stingers, in turn decreasing the density of corpses again.

## Model Design Principles

We followed and extended the approach on model design of Gilpin and Ayala [41], who formulated four principles that should be considered in model building: Simplicity, Reality, Generality and Accuracy. These principles were elaborated to establish our model design objectives, which we formulate also along four principles:

1. Objective 1 (“The principle of robust processes”): This principle sets the goal to develop a model that depicts the underlying processes of task partitioning in *E. ruidum* at a level of detail that no computational or other artefacts emerge when the value of a single basic parameter is changed to a different but still plausible value. For example, increasing the rate of recruitment to one task or changing the running speed of an ant should not lead to negative or inplausible values of focal system variables. We highlight the significance of this principle by an example: All previously published mathematical models on the collective foraging of *Ectatomma ruidum* work well in the studied range of parameters, which is also the range of parameters the observed natural processes have. However, these models will not predict plausible results anymore for a similar process that operates with different speeds (like larger or faster ants) as we will demonstrate in our study. This is especially significant when modeling self-organization and collective self-regulation of social insects is implemented as bio-inspiration of technical appliances. While the domain of bio-inspired and bio-mimicking engineering likes to retrieve inspiration form biological and mathematical models, these models have to be very general and robust. For example, the properties of a robot and those of an ant differ significantly. However, research on swarms of robots could benefit from the self-regulation mechanisms found in the ants, if a general and robust model is available. In addition to that, comparative studies (e.g., self-regulation of ants versus wasps) that aim to find common properties of collective behaviors require general and robust models in spite of the fact that biological species often differ significantly concerning individual parameters and life histories (e.g., worker size, speed, running versus flying). We also stress and test that the model’s predictions should also be stable against external disturbances of food income or worker loss, as these phenomena frequently happen in natural systems.
2. Objective 2 (“The principle of shared substances”): This principle sets the goal to develop a model that allows the global system being driven by shared substances accumulating in common stomachs. Such a common stomach is a shared compartment that is accessible for all workers and that is used to deposit foraged substances and to retrieve these substances from there whenever needed. The common stomach will saturate in times when the sum of all influxes is higher than the sum of all outfluxes. Analogously, it will get empty when the sum of all outfluxes is higher than the sum of all influxes. The current state of of the common stomach affects important components of the system, such as the recruitment rates for specific tasks. Modulation of behavior based on the common stomach is reported from many social insect species, e.g., wasps that share water by trophallaxis [29], [42], honeybees that share nectar and brood food via trophallaxis [43], [44], [45]. In ants, Cassil and Tschinkel [46] reported food sharing by trophallaxis. One important property of “common stomach”-driven regulation of work is that these systems ensure the conservation laws (e.g., conversation of mass) and therefore it is easy to implement empirical measurements into modeling of such systems. Modeling of such systems should suggest empirical quantitative experiments that will allow to observe saturation and depletion effects in the real system and in their corresponding models. The “common stomach hypothesis” is an alternative approach to explain task partitioning in insect societies without requiring non-linear (often sigmoid) threshold curves as they are suggested in many other modeling approaches [47], [48].
3. Objective 3 (“The principle of localized interaction”): This principle sets the goal to develop a model that stresses the importance of local interactions of workers with each other and with the environment. We intend to construct the model to be consistent with existing knowledge of proximate mechanisms in predator-prey systems in general and with the ant foraging system of *E. ruidum* in particular. Schatz [27] showed that the number of stingers positively correlates with the prey density, in contrast to that the model of Theraulaz et al. [38] uses the numbers of prey to modulate the recruitment of stingers. This solution worked for the model of Theraulaz et al. [38], because the authors kept the area of the hunting site in their simulation studies constant. However, Schatz [27] gave empirical results of different prey numbers and various sizes of the hunting area, thus this study investigated different levels of prey density. In consequence, we deliberate, that implementing prey density as a key model variable will lead to a significant improvement compared to the previously published models. This is also consistent with the classical understanding of predator-prey interactions [49]. If an agent can interact with another object only in a limited local neighborhood then this agent has to meet this object to allow for interaction. In a top-down modeling approach this is best described by a density-dependent probability function.
4. Objective 4 (“The principle of natural assumptions”): This principle sets the goal to develop a model that depicts a set of more natural situations and is not bound to a special experimental setup. For example, we assume a steady influx of prey into the foraging area, in contrast to a fixed number of prey as it is implemented in the experiments of Theraulaz et al. [38]. Natural environment is commonly fragmented and it has an important role in predator-prey dynamics [50] and immigration. Schatz [27] has shown that, in the case of *Ectatomma ruidum*, the maximum number of hunters that are recuited depends on the colony’s size: This relationship is also important to be implemented in order to extend the scope of the model and to keep the model as close as possible to a real system.

## The Model

### 1. Implementation and construction of the model

**Figure 2:**
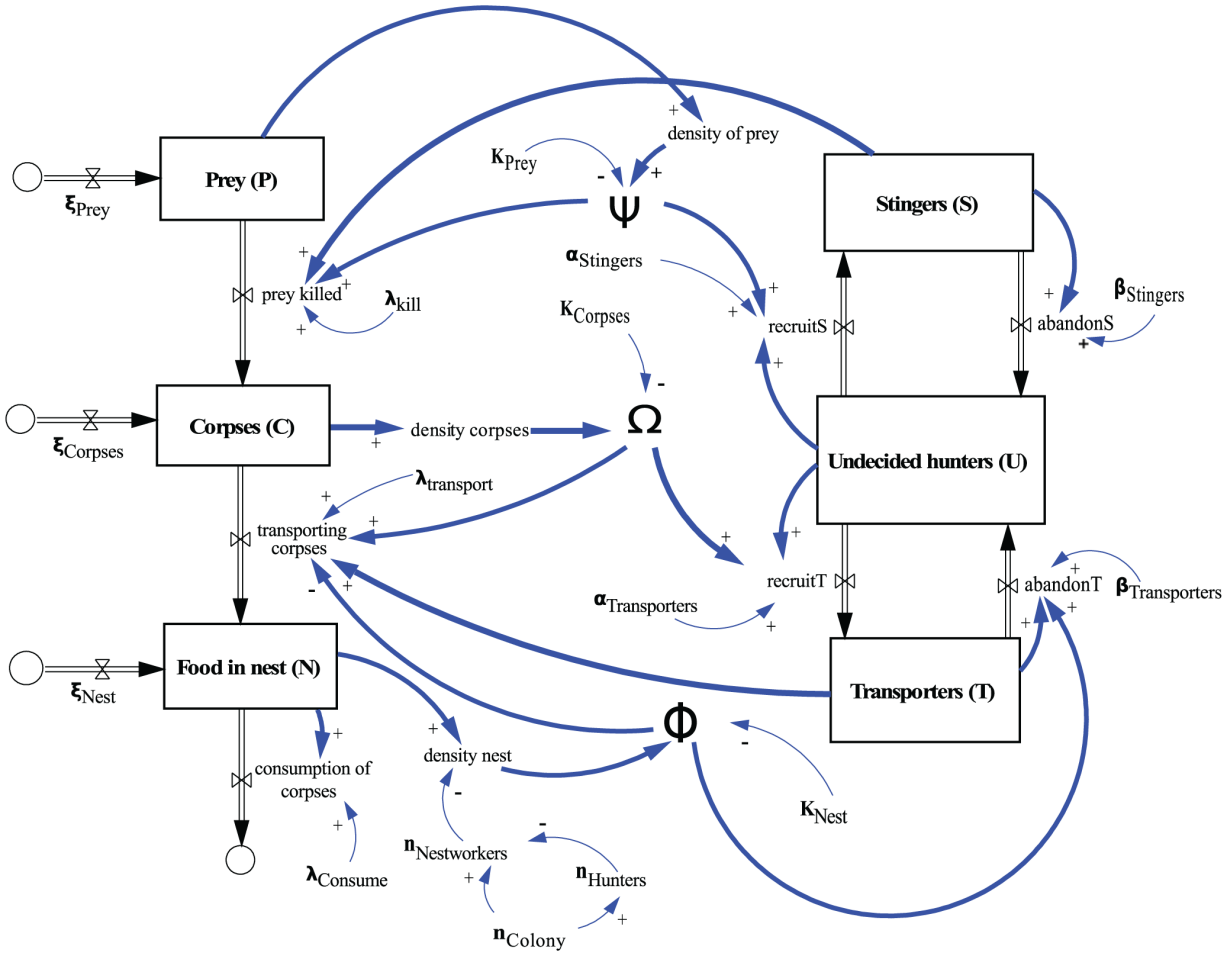
Stock & flow representation of the model of task partitioning of *E. ruidum.* For further details, please see text in section called Implementation and construction of the model.

Based on the principles mentioned before, we constructed our model on two different software platforms. We first chose a “Stock & Flow” representation of the system by implementing the model in Vensim [51], following the school of “system dynamics” [52], [53], [54], [55]. This modeling technique allows to depict material flows and accumulation of this material in stocks in a very visual way. System dynamics models ensure the conservation of mass, which is a very important aspect in our model (Objective 2). The model consists of 6 stocks, 10 flows and 19 variables/parameters (Fig. 2, Table 1). The thick blue arrows indicate the most important interactions between system components, as these interactions establish feedback loops that control the system’s global behavior by considering the current saturation value of the common stomach. Boxes (stocks) indicate compartments that hold quantities of ant workers or prey/corpse items. These compartments indicate the status of the agents they represent: stinger ants (*S*), undecided ants (*U*), transporter ants (*T*), live prey (*P*), dead corpses still outside the nest (*C*), dead corpses already transported to the nest (*N*). The numbers of ants in these states are the major system variables of our model. The doubled arrows (flows) indicate “rates of change” by which quantities of the agents can change from one stock to another stock per time period. All state transitions are modeled this way. All other (single-lined) arrows indicate interactions, meaning that a change in the numerical value of one component can induce a change of the value of another system component, whereby “**+**” symbols and “**−**” symbols at the arrowheads indicate whether this correlation is negative or positive. Finally, the circular symbols indicate sources and sinks through which quantities can enter or leave the system in a controlled way, thus these symbols indicate our system boundaries. From the position of the sinks and sources it is clear that the subsystem of prey dynamics is modeled as an open system, but the population of ants is modeled as a closed system.

**Table 1:** Parameter values and their literature sources.

variable/parameter	value	Unit	Description	source
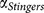	0.06	Ants	Recruitment rate for stinging	1
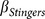	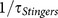	1/min	Abandonment rate for stinging	1
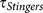	33	Min	Mean time period of stinging	1
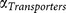	0.04	Ants	Recruitment rate for transporting	1
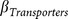	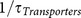	1/min	Abandonment rate for transporting	1
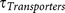	48	Min	Mean time period of transporting	1
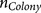	500	Ants	The total colony size	3
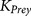	7	prey/cm^2^	Saturation capacity for prey	2
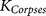	7	prey/cm^2^	Saturation capacity for corpses in the environment	2
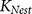	0.1	prey/ant	Saturation capacity for corpses in the nest	4
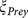	0.25	prey/min	Influx (immigration) of prey	3
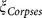	0	prey/min	(Experimental) influx of corpses	3
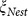	0	prey/min	(Experimental) influx of corpses into the nest	3
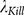	0.33	prey/ant/min	The rate at which prey is killed in case of *P(t) = K_Prey_*	1
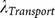	0.22	prey/ant/min	The rate at which corpses are transported to the nest in case of *C(t) = K_Nest_* and *N(t)* = 0	1
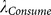	0.01	1/min	The fraction of food in the nest that is consumed per minute	4
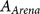	52.8	cm^2^	The default size of the hunting area.	1, 2

Sources: 1: Theraulaz et al., [38]; 2: Schatz et al., [27]; 3: parameter values used for experiment with our model; 4: our assumption.

In the next step we implemented the model in an ODE-based modeling platform called SAGE [56]. We lost the visual connections of the variables by using this implementation form, but some of the calculations and simulations were faster and easier in SAGE, allowing thus an exhaustive model analysis. The re-implementation of the model includes describing the dynamics of each stock as a separate ODE, having the inflows as positive and the outflows as negative terms in the RHSs of the equations. As the flows link the stocks in the stock&flow model (see Fig. 2), the resulting ODEs in SAGE form a system of first-order ODEs which was solved numerically by 4^th^-order Runge-Kutta-Method.

### 2. Modeling task partitioning of hunting behavior

As a first step to our model, we describe the dynamics of the three task groups of ants that participate in the task partition of hunting behavior. Each individual ant can be in one of the possible 3 behavioral states. The initial state of all ants is the state ‘undecided’ *U(t)*, what means that they are neither specialized to sting nor to transport. If such an undecided ant meets a living prey animal in its local environment, it stings (thus kills) this prey and thus becomes a stinger ant (*S(t)*) for some time (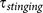). After this time period it stops stinging and becomes an undecided ant *U(t)* again. As a conservative assumption we assumed randomized movement of the undecided ants and also a random distribution of prey items (*P(t)*), thus we followed the expressions of classical Lotka-Volterra-type predator-prey models, which also use mass-action law to model the interactions between predators and prey. We assumed that the higher the spatial density of prey is – what is expressed by our first “common stomach” variable Ψ(t) – the faster an undecided ant encounters a living prey item and the faster (and thus more likely) it is recruited to the stinging task. This process of recruitment for stinging is therefore modeled as *α_Stingers_* ⋅Ψ(*t*)⋅*U*(*t*) whereby 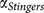 is the recruitment rate of undecided workers to stingers. The abandoning from the stinging task is expressed by 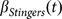, whereby 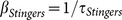 is the abandonment rate of stinging ants. These considerations finally lead to equation 1, which describes the dynamics of the stinger ant task group:
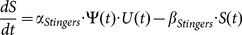
(1)


The dynamics of the third hunting task, which are the transporter ants (*T(t)*), are modeled in a similar way, with the only exception that we assumed that the recruitment of undecided ants to the transporting task is positively correlated with the density of prey corpses (*C(t)*) in the environment. This relationship is represented by the saturation variable of the corpse-associated common stomach (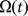) and a given recruitment rate to the transportation task (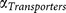), leading to *α _Transporters_* ⋅Ω(*t*)⋅*U*(*t*) for describing the dynamics of the recruitment process of transporters. The abandonment of ants from the transportation task is assumed to be correlated positively with the density of corpses in the nest as it is expressed by our nest saturation common-stomach variable (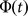) as well as with a fixed abandonment rate 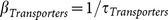 leading to *β_Transporters_* ⋅Φ(*t*)⋅*T*(*t*) as a final model of the abandonment process of transporters.

In short, we assumed that corpses are distributed randomly in space and that transporter ants move randomly while they search for corpses to pick up and while they search for delivery places of their carried corpses in the nest. The phase of directed motion towards the nest was neglected in our model, except in the timing setting of 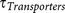. Thus, we assumed that higher corpse density in the environment leads to more frequent encounters of already stung corpses by undecided ants. Thus, more ants are recruited per time unit to the transporting task in times of higher corpse densities. In addition we assumed that the more corpses are already gathered in the nest the longer will take a transporting ant to find a receiver worker or larva in the nest. Therefore the longer such an ant has to search for a receiver, the more likely it will abandon the transportation task. In equation 2, these considerations are summarized to describe the dynamics of the transporter task group.
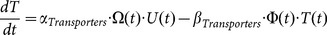
(2)


In our “common stomach” model approach it is essential that the modeling does respect the conservation laws for quantities, masses of substances and the conservation of energy (Objective 2). Thus, as we consider our ant worker population as being a closed system we can model the dynamics of the undecided workers by the following equation 3, whereby 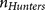 expresses the total number of hunting ants and *U*(*t*) expresses the number of undecided ants:
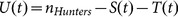
(3)


**Figure 3:**
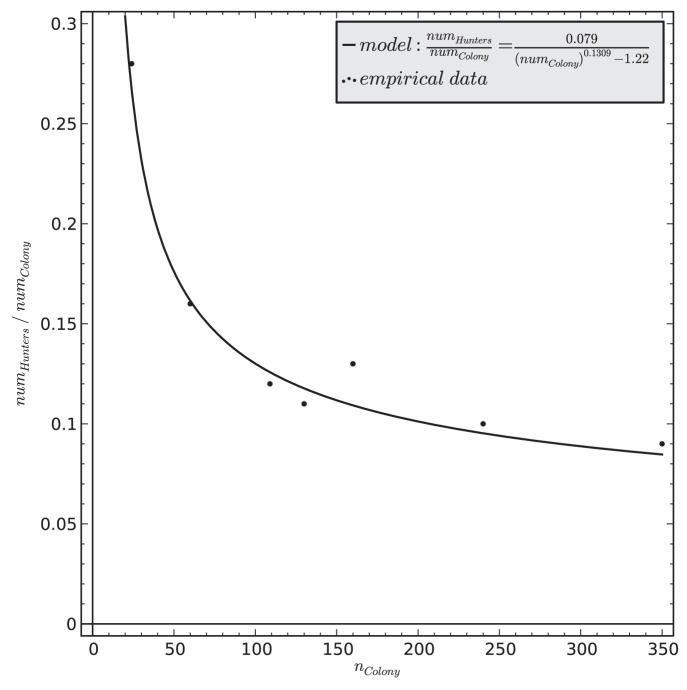
The model of predicting the fraction of actively hunting ants as a function of the total population of the ant colony (black line). The circular data points indicate empirical observations of Schatz ([27], Fig. 1B in their paper). The presented curve fitting yields a summarized squared error of *ε* ^2^ <0.0007.

As it is pointed out in Schatz et al., [27], the fraction of ants that engage in the hunting task in the full colony population (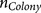) depends on the colony size. He found that smaller colonies have a higher fraction of workers engaging in hunting than larger colonies. By using optimal fitting technique we found the best fit for Schatz et al., [27], Fig. 1B in their paper) data described by the curve 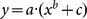 to the field data (Fig. 3 and equation 4).
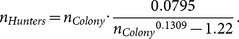
(4)


In our model, the dependence of the number of hunters on the colony size is defined between 10 and 1000 workers only. This is the range in which Schatz et al., [27] presented field data and also the range of colony size in which our model provides predictions. Colonies with fewer than 10 individuals would have implausible fractions of hunters in the colony predicted by our equation 4. This limitation is due to that fact that no empirical data on smaller colonies exist to be used in the model fitting of this curve. This, in turn, can be reasoned by the assumption that in natural conditions such colonies might not use task partitioning for their hunting (see Objective 4) at all.

### 3. Modeling the common stomachs

In our modeling approach we assumed that the living prey, the corpses in the environment and the corpses in the nest change their spatial densities over time, as the ants convert and transport these items (Objective 2). It is reported by Schatz et al., [27] that the density of prey affects the recruitment of stingers. They also observed the highest number of stingers with a density of 8 prey/cm^2^. Lower densities of prey (7 or less prey items per cm^2^) on the hunting area resulted in lower numbers of recruited stingers. Based on these data we assumed a value of *K_Prey_ = 7 prey/cm^2^* which is able to trigger the maximum recruitment of stingers. In a similar way, we assumed *K_Corpses_ = 7* prey/cm^2^ to be the density of corpses in the environment that is able to trigger the full recruitment of transporters. While *K_Prey_* and *K_Corpses_* are based on a maximal spatial density of prey items, we assumed that the saturation of the nest with corpses is bound to the colony size. We assumed that a fully saturated nest is represented by a *K_Nest_ = 0.1* prey/ant, meaning that one prey item can saturate/occupy 10 nest worker ants. To model the common stomach saturation, we first modeled the density of the system variables for prey *(P(t)*), corpses in the environment (*C(t)*) and corpses in the nest (*N(t)*) in the following equations 5, 6 and 7:
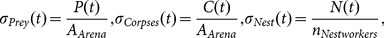
(5,6,7)where *A_Arena_* is the size of the hunting arena and *n_Nestworkers_ = n_Colony_ – n_Hunters_* is the number of the workers that don’t engage in the hunting process but do engage in other tasks, including: taking care of the brood or the queen, nest building or colony defense. We would like to point out that our model follows the classical ecological models that incorporate density-dependent growth and saturation processes, as it was described and modeled for example by J. P. Verhulst [57] and which were later incorporated by the classical models of interspecific competition by Volterra [58]. The values of *K_Prey_, K_Corpses_ and K_Nest_* can be seen as limited capacities of the system for prey and corpses, thus as soon as these populations reach these maximum capacities the corresponding rates of change reach their extreme values (**0** or **1**). Prey or corpse densities between **0** and **K** are predicted to produce only linear changes of these rates of change. The observed non-linear system behaviors stem from the interactions among components which form feedback loops but do not exploit already built-in non-linearities, as it is the case in threshold-based models and also in most agent-based models (Objective 3 and 4).

Based on these densities we could model the current saturation of the 3 common stomachs by the following equations 8, 9, and 10:
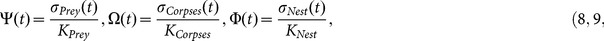
(8,9,10)


### 4. Modeling material flows

The detailed modeling of the associated material flows is a fundamental issue concerning our hypothesis, whether or not task partitioning can be regulated by a shared material-based common-stomach. The first important process to model is the rate with which the stingers kill prey and convert them into corpses which are then deposited in the very same area. In accordance to classical predator-prey models and to empirical experiments reported by Schatz et al., [27] on *E. ruidum*, we assumed that the stinging productivity scales (positively) with the density of prey. Thus it also correlates with the saturation of the common stomach of prey, as it is expressed by 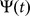. The rate of the stinging process is also assumed to increase with increasing numbers of stingers (*S(t)*) and with a constant factor of 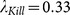 prey/ant/min, which is reached only when the common stomach of prey is fully saturated, that is whenever 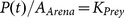 and thus 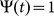. In conclusion, the amount of killed prey per minute is given by λ*_Kill_* ⋅Ψ(*t*)⋅*S*(*t*).

We also assume that prey does not reproduce within the timescale of our model (from a few hours up to one day). Thus, we only have to consider an immigration influx of prey (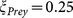 prey/min) as well as a starting population of prey *(P(0) = 0* prey in most experiments). These formulations lead us to equation 11, which describes the dynamics of prey items:
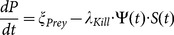
(11)


The second common stomach is conceived as the number of corpses that are located in the environment and which are waiting for transporters to be taken to the nest. In our system this second common stomach is the most important one, because it is regulated on both sides, i.e., its infux and its outflux are precisely controlled by the working ants. The number of corpses increases with the influx of killed prey (see last term of equation 11). In parallel, it decreases with the flux of corpses that are transported from the hunting site to the nest by transporter ants. We consider corpses to be in the nest only after the transporter successfully handed the corpse over to nestmates or by dropping the corpses at a suitable place at the deposition site of the nest. We assume that this rate depends on the number of transporters, on the saturation of the environment with corpses (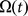) and on the saturation of the nest with corpses (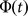). On one hand, higher density of the corpses in the environment result in quicker encounters of corpses by transporter ants, thus the transportation rate will increase with increasing density of corpses. On the other hand, if corpse density is high, the nest can in consequence saturate with corpses and this, in turn, will increase the time it takes the loaded transporters to finally deposit their corpses. Thus transport rates will go down as the nest saturates with corpses or the environment gets emptied of corpses. In analogy to the model of maximum killing rates, we assumed that the highest transportation rate of 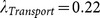 prey/ant/min to be reached if 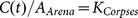 and thus 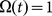 and 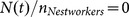 and thus 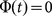. Therefore, the rate at which corpses are transported from the environment to the nest can be described as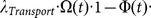 *T*(*t*) and the full dynamics of corpses can be modeled by the following equation 12:
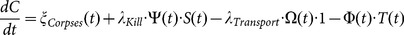
(12)


To describe the dynamics of the corpses inside the nest (*N(t)*) we had to consider several flows of material. First, we considered the influx of corpses carried to the nest by transporters described by the second term of (12). We assumed that the corpses are consumed over time in the nest (outflow) with a constant rate, which we set to 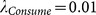 per min, this means that we assumed that the average time they spent in the nest before they are consumed is 100 min. This gives the final equation of the dynamics of the corpses stored in the nest:
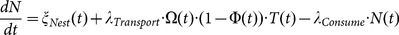
(13)


We also implemented external or experimental additional influxes into the common stomachs formed by prey 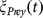, (simple addition to the immigration), by corpses in the environment 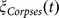 and by corpses in the nest 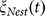 as constant rate fluxes, similar to our model of the immigration of prey. These rates allow to model experimental scenarios (for example experimental addition of prey or corpses to the environment or nest). Generally we set these experimental influxes to zero, exceptions are noted otherwise.

### 5. Measuring the predicted efficiency of the colony

The whole foraging process has the purpose of bringing nutrients (corpses) into the nest to feed the workers, the queen and the brood. Therefore, it is important to measure the efficiency of the collective foraging. We argue that the transportation rate of the corpses into the nest is a good metric for this efficiency, significant for the ecological and evolutionary interpretation of this natural system of task partitioning and decentralized coordination. However, such a measure has to consider the colony size, as well. Larger colonies have more hunters and transporters to collect food. It is also important to describe such collective systems by their per capita efficiency, as often synergetic effects and beneficial or detrimental effects relate to colony size are mentioned as ultimate reasons for the emergence of such collective behaviors [13], [31], [3], [59]. Thus, we define the efficiency of the process 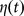 for every time step **t** of the process by our model equation 14:
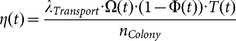
(14)


To express the total efficiency over time and with various colony sizes, we also used an accumulated fitness measure 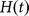, which summarizes the values of 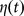 over all time steps.


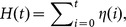
 (15).whereby 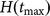 expresses the final cumulative efficiency per worker for a full simulation run.

## Results and Discussion

### 1. General patterns of collective behavior predicted by our model

Before we analyze our model in detail we want to compare the predicted results of our model with empirical data reported in literature. Schatz el al. [37] and Theraulaz et al. [38] presented a series of experiments in which the authors measured the number of transporters and stingers as well as the number of prey animals and prey corpses. These variables were measured as a function of colony size and as a function of prey population size at the hunting site. They showed that when a vast amount of prey was provided the colony was unable to process all prey items and an equilibrium group size emerged for for stingers and transporters. Such an ‘ad libitum’ condition can be investigated with our model by assuming biologically more plausible circumstances, in which living prey animals appear at a steady rate (influx through immigration) at the foraging site. To imitate such a setup we used the following setting: *P(0) = 0, C(0) = 0, N(0) = 0, S(0) = 0, T(0) = 0, U(0) = n_Colony_ = 500*, 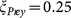, 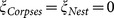, for other parameter values we used the standard setting (see Table 1).

**Figure 4:**
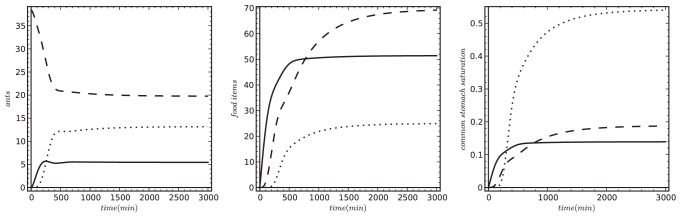
Equilibria of worker group sizes (left), food stocks (middle) and common stomach saturation levels (right) that emerge when the model is parameterized with a steady prey influx. For more details, see text. Left figure: Solid line: *S*, dashed line: *U*; dotted line: *T*. Middle figure: Solid line: *P*, dashed line: *C*; dotted line: *N*. Right figure: Solid line: 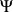, dashed line: 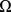; dotted line: 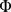.

Similar to the experimental results of Schatz et al. [37] and Theraulaz et al. [38], the system converges to stable equilibria of task group sizes (Fig. 4 left), food stocks (Fig. 4 middle) and saturation levels of the common stomachs (Fig. 4 right).

**Figure 5:**
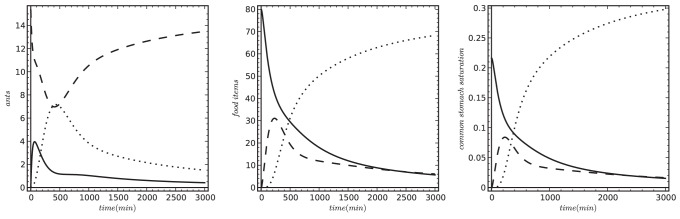
The predicted colony reactions to a small number of prey (*P(0) = 80)* provided at the start of the experiment without further prey influx leads to a pulsed response of the colony. Our model predictions show a striking similarity to empirical data of a comparable experiment described in Theraulaz et al. [38] and Schatz et al. [37]. Left figure: Solid line: *S*, dashed line: *U*; dotted line: *T*. Middle figure: Solid line: *P*, dashed line: *C*; dotted line: *N*. Right figure: Solid line: 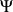, dashed line: 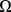; dotted line: 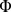.

In a different setting, Theraulaz et al. [38] showed that when the authors supplied only a small number of prey to a colony without any steady influx/immigration of prey, the colony reacted with a quick peak of foraging activity. Soon after that the environment was depleted from prey and this in turn led to a cessation of foraging activities. Our model predicts similar collective behaviors (Fig. 5) when parameterized with *P(0) = 80, C(0) = 0, N(0) = 0, S(0) = 0, T(0) = 0, U(0) = n_Colony_ = 130 ants*, 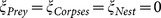 *prey/min*, other parameters were set as it is shown in Table 1 **.** In case of such a small colony size and artificial prey influx it is unclear how the nest saturation can affect the foraging at short timescales. Thus, we practically eliminated these nest-saturation effects from our runs by setting *K_Nest_ = 2* and 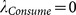.

Our modeled colony reacts qualitatively similar to the experimental data of Theraulaz et al., [38], but in a slower pace. This is due to the fact that we used the values reported by Theraulaz et al. [38] for the parameters 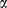, 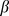, and 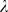-parameters although we multiply these rates additionally with the saturation levels of one or several common stomachs (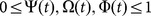), thus our model in fact uses lower recruitment, abandonment and food processing rates what slows down the overall system. The predicted responses could easily be made faster by setting the values of 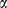, 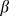, and to higher values, but for these experiments shown here we wanted to retain the parameter values that are estimated in Theraulaz et al. [38].

In a different study Schatz et al. [27], a colony of *240 ants* was confronted with two different experimental series to investigate whether the ant colony considers the total prey number or the prey density in its hunting recruitment strategy. In the first experimental series, the initial number of prey items *(P(0) = 400 prey*) was distributed in hunting areas of different sizes. This way hunting areas with 5 different prey densities were generated in a range from *1.5 prey/cm^2^* to *7.9 prey/cm^2^*. The authors reported that the colony responded to this pattern by recruiting different numbers of stingers: The higher the prey density was, the more stingers were recruited. In the same study, Schatz et al. [27] performed also a second experimental series in which they varied the number of introduced prey items from *80 ants* to *400 ants* and also adjusted the size of the hunting area in parallel in order to keep the spatial prey density constant at *1.51 prey/cm^2^*. As a result of these experiments the authors reported that the number of recruited stingers did not change with the increasing number of prey, but with increasing prey density.

**Figure 6:**
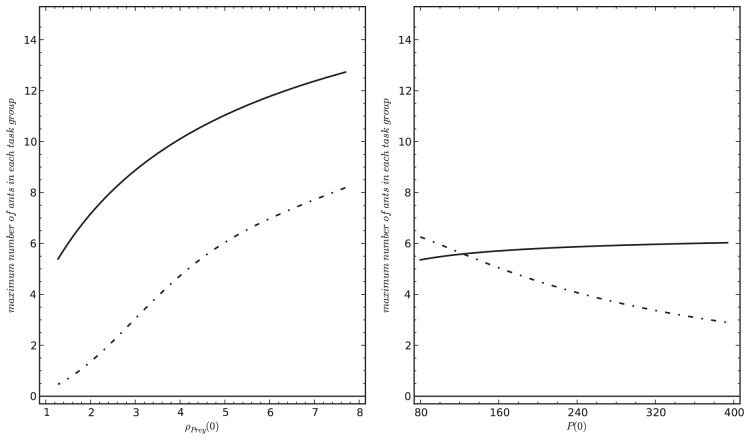
Predicted response of the ant colony to a varying density of prey items with constant total prey number (left figure) and to a varying number of prey items at a constant prey density (right figure). In both cases we measured the maximum number of stingers (solid line) and of transporters (broken lines). In both cases we used the default parameters listed in Table 1, except *n_Colony_* = 240, *t_max_* = 120. The varied parameter is indicated on the x-axis of both figures. Left figure: P(0) = 400; right figure: P(0) is varied between 80 and 380 prey items across the runs.

One of the important objectives of our model (objective 2) was to test if a “common stomach”-based model would provide comparable prediction to data reported by Schatz et al. [27]. Thus we ran our model with the same densities and numbers of prey as it is described by Schatz et al. [27]. We report the maximum peak of stingers in each setting, because such experimental settings can produce a pulsed response of the colony (Fig. 5). Our model predicted a very similar picture compared to the findings of Schatz et al. [27]: The number of ants engaged in stinging and transporting is significantly modulated by prey density, but it did not change significantly with varying number of initial prey number (Fig. 6.).

**Figure 7:**
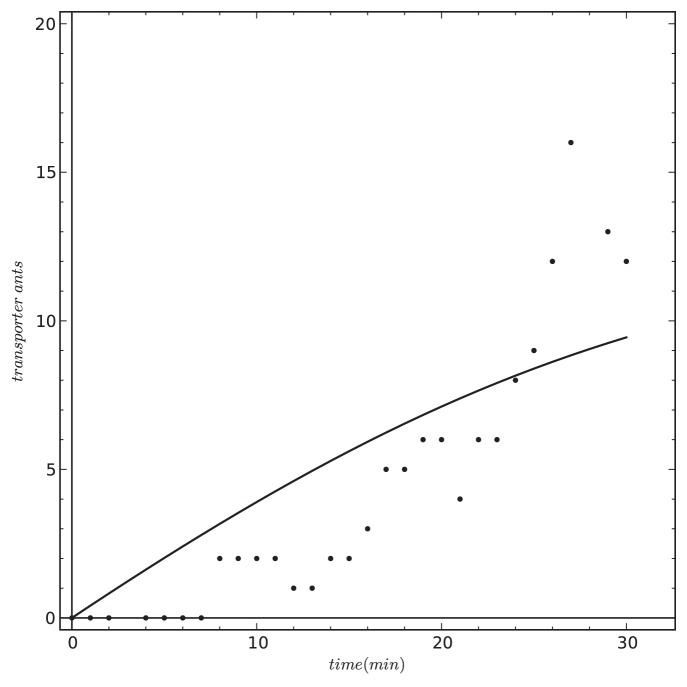
Predicted increase of the number of transporter ants of our model (solid line) to the data given by an empirical study (dots: data redrawn from Pratt [60]. Our model and the empirical study show an increase from 0 to approx. 10–12 ants over 30 minutes of the experimental run.

In order to further validate the parameters of our model, we compared our model predictions to another set of empirical data [60]. To perform this analysis, we initialized our model without initial stingers and transporters (*S(0) = T(0) = 0 ants*), no prey and corpse influx (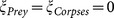 *prey items/min*), no initial prey items *P(0) = 0 prey items*) and with 100 corpses in the environment (*C(0) = 100 prey items*). The colony size was set to *n_Colony_ = 120* ants and all other parameters were set to their basic value (Table 1). The model, similar to the findings of Pratt [60], predicts an increase of transporters in the system approaching 10 transporter ants after 30 minutes of runtime (Fig. 7). It noteworthy that this set of empirical data was neither used for building our model, nor was it used for comparison/validation of the previously published model by Theraulaz et al. [38].

### 2. Analysis and discussion of the model

**Figure 8:**
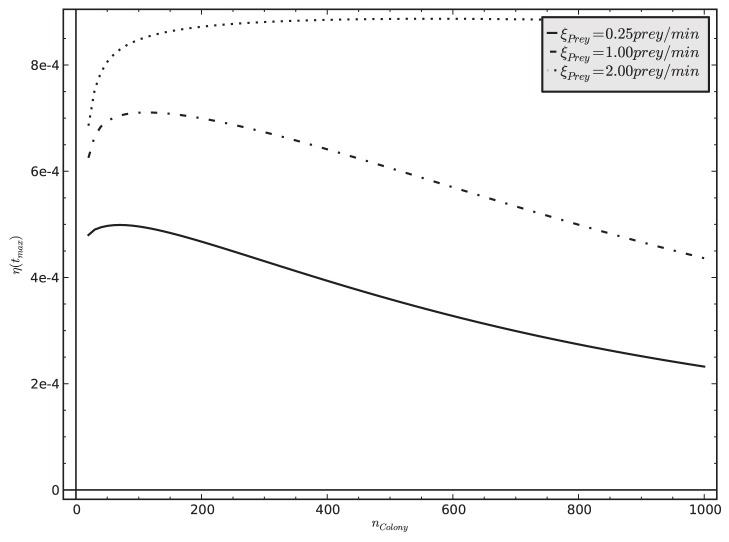
Relationship between colony size *n_Colony_* and colony-level efficiency 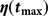 in three different prey influx 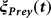. These results indicate the existence of an optimal colony size for a given prey influx rate, showing that richer prey influx allows larger colonies to operate at their optimal level.

In our parameter sweeps (S1 Text) we identified two parameters, *n_Colony_* and *A_Arena_*, that were most significantly affecting the model’s predicted dynamics of our key system variables *S*, *T*, *U*, *P*, *C*, and *N* (Table 2). To analyze how significantly these two key parameters affect the efficiency of our modeled ant colony, we varied these two parameters at 3 different levels of prey influx *(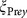 in {0.25, 1.00, 2.00} prey/min*). The model predicts an optimal colony size for different levels of prey influx. This sweet-spot shifts towards larger sized colonies as the prey influx to the hunting site increases (Fig. 8). After some transient period and without disturbance and with steady prey influx, the value of 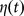 is converging to an equilibrium value, which we read at *t_max_* as 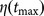. Colony size is affecting this efficiency measure at three points in our modeled systems: (1) Larger colonies can have more stingers and transporters, thus they can kill and collect more prey item per time unit. (2) Larger colonies have more nest workers, thus the nest can handle more corpses per time unit and saturates only with higher numbers of corpses in the nest. (3) These two positive effects of larger colony sizes can be cancelled out by the fact that we calculate the efficiency per worker, as every worker represents also costs for the colony. Thus, the efficiency of the colony as it is expressed by 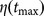 will only increase if prey density increases also proportionally, so that workers always will be able to find enough prey to sting and enough corpses to transport. In conclusion, the observable peak values of 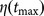 at a specific value of *n_Colony_* (Fig. 8) represent the payoff points of these above-mentioned antagonistic factors at a given level of prey influx.

**Table 2:** Parameter sweeps of model parameters.

Parameter	System variable						
	*S*	*T*	*U*	*P*	*C*	*N*	Impact of parameter
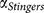	**+ +**	** = **	**− −**	**− − −**	**+ +**	** = **	**9**
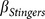	**− −**	**−**	**+ +**	**+ +**	**− −**	**− −**	**11**
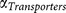	**−**	**+++**	**− − −**	**+ +**	**− − −**	** = **	**12**
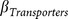	**+**	**− −**	**+ +**	**−**	**+ +**	**−**	**9**
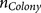	**+**	**+ +**	**+++**	**− −**	**∼ ∼**	**+ +**	**16**
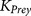	**∼**	**∼**	**+ +**	**+++**	**− −**	**− −**	**15**
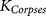	**+**	**− −**	**+ +**	**−**	**+++**	**− −**	**11**
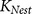	**−**	**+ +**	**− −**	**+**	**− −**	**+ +**	**10**
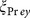	**+ +**	**+ +**	**− − −**	**+ +**	**+++**	**+**	**13**
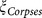	**−**	**+**	**−**	** = **	**+**	**+**	**5**
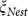	** = **	**−**	**+**	** = **	**+ +**	**+ +**	**6**
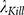	**− − −**	**+ +**	**∼**	**− − −**	**+ +**	**+ +**	**15**
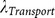	**+**	**− − −**	**+++**	**−**	**− − −**	**+ +**	**13**
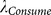	** = **	**+ +**	**− −**	**+**	**− −**	**− − -**	**10**
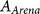	**−**	**∼**	**∼**	**+++**	**∼ ∼**	**− −**	**18**
**Sensitivity of variable**	**20**	**29**	**34**	**25**	**41**	**24**	

Summary of the reaction of the core system variables to changes of selected model parameters. Symbols: “∼”: complex reaction without change of the type of correlation, “∼ ∼”: complex reaction exhibiting multiple types of correlations (positive and negative), “+”: increase, “+ +”: significant increase (up to 50% of maximum variable range), “+++”: very strong increase (50% to 100% variable range), “−“: decrease, “− −“: significant decrease (up to 50% of maximum variable range), − − −: very strong decrease (50% to 100% variable range), “ = ”: no considerable change. The column “impact” indicates how sensitive the model reacts to changes of each parameter and the row “sensitivity of variable” indicates how strongly each given state variable was affected in total by these parameter changes. Values for these measures are calculated as sum of signs (except “ = ” that counts as zero and “∼” to be equivalent to 3 indicating a complex reaction).

**Figure 9:**
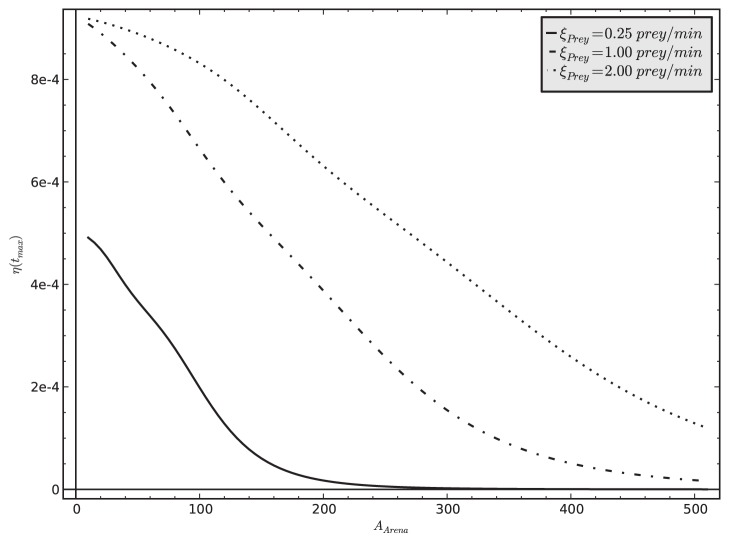
Relationship between the size of the hunting site *A_Arena_* and the colony-level efficiency 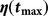 in three different prey influx 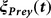 conditions. The results indicate that larger hunting sites have a lower prey density at a given prey influx level, what makes hunting and transportation less efficient, what in turn negatively affects the colony-level efficiency metric.

On the other hand, an increase of *A_Arena_* shows a negative correlation with the colony-level efficiency 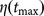, see Fig. 9). A stronger influx of prey allows the colony to increase its predicted colony-level efficiency, indicating that rich environmental prey supply can be efficiently exploited by the colony.

### 3. Perturbation experiments

Performing perturbation experiments with our modeled system makes it possible to test the robustness of the system and to generate novel testable hypotheses that can be tested in future field or lab experiments. To construct such testable hypotheses with our model, we increased or decreased the influx of materials that flow into different common stomachs in the form of a sudden perturbation that lasts for a given period of time. Such perturbations can be performed in empirical experiments by simply adding or removing prey animals or corpses to/from different locations (hunting site or nest). In each case the model was run until its system variables stabilized and then one system component after the other was perturbed suddenly. In a natural system the adaptation to such sudden changes requires some time to process, therefore perturbation were not applied as one short instant only. Instead, the perturbed parameter was kept on its perturbed value throughout 500 *min.* For example, after the system stabilized at time step *t_max_ = 500 min* we increased the prey influx from its standard value (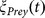 *= 0.25 prey/min*) with a constant additional influx by increasing 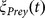 *to 0.45 prey/min* for *500* *min*, then the perturbed parameter was set back to its standard value. After this, the system stabilized again and a similar experiment was carried out. This time the prey influx was decreased at time step *2500 min* with a constant value for a period of *500* *min* and then this perturbed parameter was again set back to its standard value.

These perturbation experiments show that our proposed model reacts in a very stable and robust way to such perturbations. This demonstrates that our first objective, the principle of robust processes, is met by our model. The collective system is reactive, because changing these variables results in a new equilibrium and our key system variables change at a lower extent than the changes we did induce before on the perturbation parameter. The system is also resilient, as we found that the system variables converge back to their original equilibria after the end of the perturbation periods. The modeled system shows a reasonable degree of change, it neither jumps to high values nor does it obtain negative or inplausible values. The model does not scale linearly to the increase of a given parameter, because strong shifts in the perturbation parameters result in under-proportional reactions of system variables compared to weak shifts. These results also indicate that the model is robust and that the system is strongly governed by negative feedback mechanisms.

**Figure 10:**
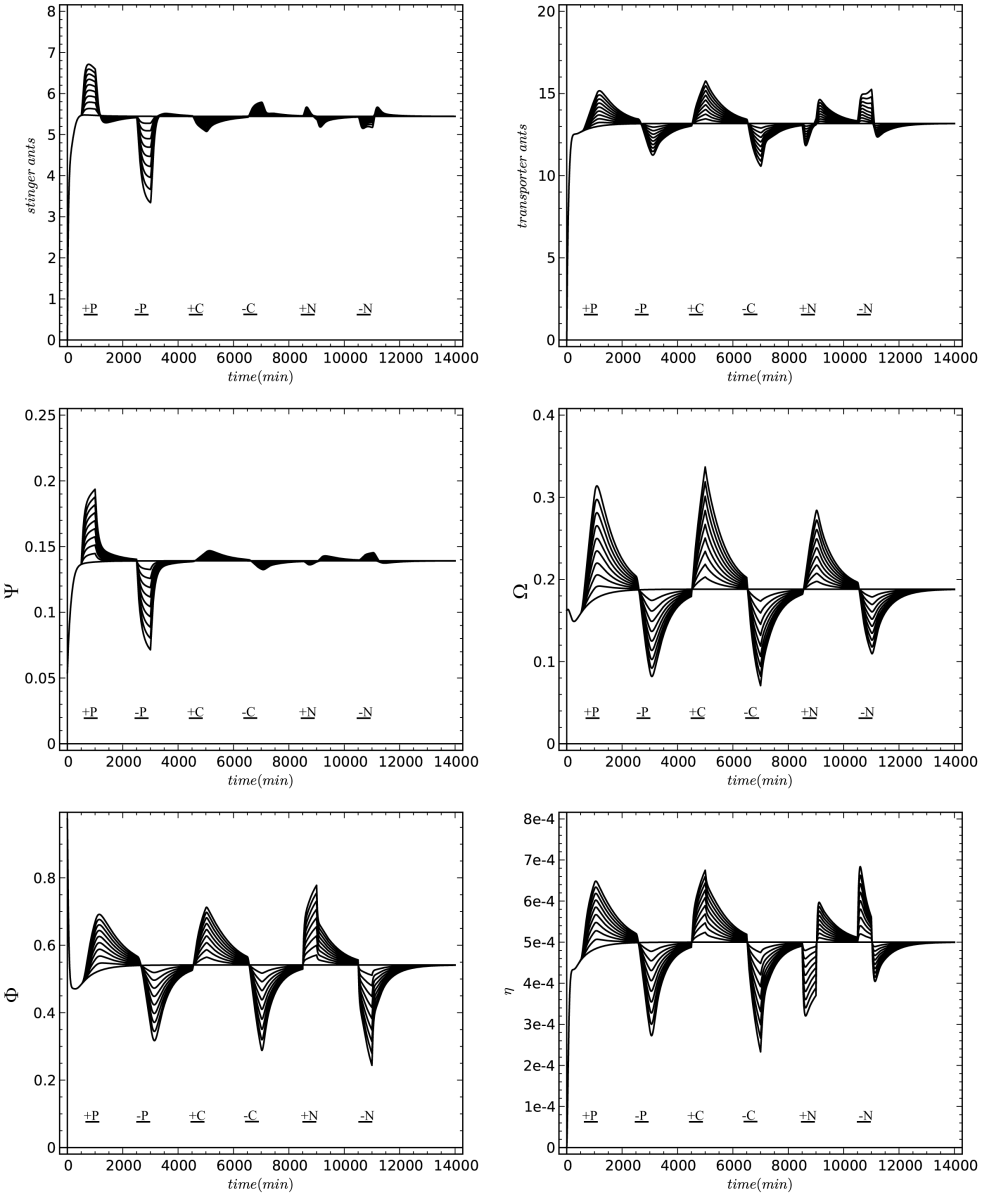
Time plots of our model’s system variables (*S, T*, Φ, Ω, Ψ, *η*) in simulations were the colony was exposed to a specific regime of perturbations in the supply of prey and corpses. Perturbations: P+: Prey influx increased (*500 min* −*1000 min*); *P*-: prey influx decreased (*2500 min* −*3000 min*); *C*+: influx of corpses increased (*4500 min* −*5000 min*); C-: corpse influx decreased (*6500 min* −*7000 min*); *N*+: influx of corpses in the nest increased (*8500 min* −*9000 min*) and *N*-: influx of corpses in the nest decreased (*10500 min* −*11000 min*). The baseline (horizontal line) shows a simulation run made with our standard parameters (see Table 1). Lines above this baseline represent runs with increased values (adding *+0.02*, *+0.04*, …, *+0.2* to the standard fluxes). Lines below the standard line represent similar simulation runs, except that the influxes were decreased at the same extent as they were increased previously.

Increasing the prey influx results in strong increases in prey and corpses at the hunting site and in the nest. Decreasing the prey influx mirror these effects (Fig. 10). A perturbation which is induced by changing the influx of corpses shows a weak effect on the stingers and on the saturation of prey densities. Furthermore it has a prominent effect on the number of transporters and on the number of corpses in the field and in the nest. However, such perturbations affect the efficiency of the colony in a significant way. Perturbing the colony by changing the influx of corpses into the nest has the weakest effect on system variables in general, except on the saturation of the nest with corpses.

Manipulating the saturation of the nest with corpses (last two perturbations) is predicted to trigger a more complex reaction by the colony (Fig. 10). As a first response to a sudden increase in the nest’s saturation with corpses, the commons stomach of corpses in the nest (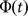) fills up and in consequence the number of transporters decreases, because the corpse-saturated nest makes it harder for transporters to deliver their load. Thus recruitment of new transporters decreases and abandonment of transporters increases (see equation 2). The lowered number of transporters causes the corpses to accumulate at the hunting site, therefore 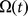
increases. In turn, the number of stingers decreases, because they are recruited less effectively to this task and abandon more often from this task (see equation 1). As a final result of these cascading effects, the efficiency of the perturbed colony (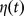) decreases, because this metric considers only those corpses that are actively delivered to the nest by the ants and neglects physical manipulation of the nest stocks.

After several hundreds of minutes the colony consumed the excess corpses that were stocked in the nest as a consequence of the perturbation. At this point of time, the numbers of transporters and stingers are lower than normal and many corpses and prey have accumulated at the hunting site. Thus, as soon as the nest is not saturated with copses anymore, the recruitment for hunting and stinging tasks increased in the second half of the perturbation pulses and several system variables changed drastically: The recruitment of stingers and transporters dropped in the first phase of the perturbations. In contrast to that, the colony recruits more stingers and more transporters than normal (Fig. 10) during the second phase of the perturbation phase. Decreasing the corpse saturation of the nest provokes a similar reaction compared to the one we described above, but with a contrasting trend: At first an increase of the number of stingers and transporters can be observed and then, after a saturation of the nest is achieved, the system swings back in the opposite direction. The complexity of these responses is produced by the fact that linked common-stomach subsystems act together in a causal network, and various time delays, e.g., accumulation of corpses at various places, affect the system’s responses in parallel.

In short, the system reacts in a very adaptive manner to the tested perturbations. System variables that are directly affected by the performed perturbations change rather strongly while other system variables, which are only indirectly affected by the perturbation of influxes, are effected in a weaker and in a more delayed manner. In conclusion, our model predicts that the experimental perturbations of influxes are propagating through the whole system and can be detected by worker ants at many places and in various interactions. The model further predicts that these disturbances also significantly affect the efficiency of the overall system.

Using an extensive sensitivity analysis of the model (S2 Text) we also concluded that the combination of different values of the paremeters will not create non plausible results and the model remains robust.

### 4. Optimization runs

In order to identify an optimal colony size for a given prey influx we performed the following analysis: We kept all parameters at their default values (Table 1) except 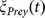, which was varied within 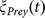 *in {0.25, 0.5, 1.0, 2.0} prey/min*. This parameter represents the influx of living prey into the hunting area, thus it is an external parameter of the ant system. We used an optimization algorithm (Powell algorithm) built into Vensim [51] to maximize the cumulative efficiency metric 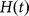 by changing the colony size (*n_Colony_*) of the ant colony. This way the optimization algorithm finds the optimal colony size for the given prey influx by searching a colony size that yields the maximum colony efficiency (Table 3). In this analysis we used 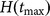 as cost function. This was considered to be the better choice for an efficiency metric for this analysis here, because we carried out no perturbations in the optimization runs, given that such treatments would have biased the dynamics of 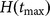 due to its cumulative nature and the perturbations would also have masked the investigated focal effects. In addition to that, the cumulative nutrient income is more important than just the final one at *t = t_max_* for predicting survival chances of colonies. The results of this optimization analysis show that doubling the influx rate of prey shifts the sweet spot of colony size in a linear manner: Every doubling of the influx rate yields an optimal efficiency at an approximately doubled colony size.

**Table 3:** Predicted optimal colony size as the function of the prey influx rate.

Optimization run:	1	2	3	4
Influx of prey:	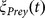 *** = 0.25 prey/min***	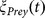 *** = 0.5 prey/min***	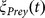 *** = 1.0 prey/min***	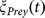 *** = 2.0 prey/min***
Max. payoff found at:	n_Colony_^*^ *** = 93*** ** ***ants***	n_Colony_^*^ *** = 182 ants***	n_Colony_^*^ *** = 360 ants***	n_Colony_^*^ *** = 702 ants***
The final payoff predicted:	*H(t_max_)* *** = 2453.5*** transported corpse per worker ant	*H(t_max_)* *** = 3037.6*** transported corpse per worker ant	*H(t_max_)* *** = 3495.9*** transported corpse per worker ant	*H(t_max_)* *** = 3834.8*** transported corpse per worker ant

The variable n_Colony_^*^ indicates the optimal value of n_Colony_ for a given value of 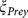, which was identified by a Powell optimization algorithm [51] for yielding the highest predicted value of H(t_max_).

Another free parameter of the model is the consumption rate of corpses in the nest (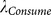), which reflects the “hunger status” of the colony. In nature this parameter is mainly influenced by several internal and external factors (season, weather, brood status, queen activity, colony size) which are mostly not part of our model. However, our model considers the colony size, which is a significant contribution to the food consumption in the colony. Thus we performed another optimization experiment by keeping the prey influx constant at a high rate of 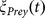 *= 1.0 prey/min* and by varying the consumption rate of corpses in the nest 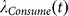 *in {0.01, 0.02, 0.04, 0.08} min^−1^*. Similar to the previous experiment (Table 3), the model predicts the shifts of the sweet spot concerning the optimal colony size in an almost linear manner (Table 4). Doubling of the nest consumption rate (per worker) results in a shift of optimal colony size by approximately −*50%* of its previous value. This indicates that higher consumption rates predict smaller colonies to work more efficiently (per worker) than larger colonies.

**Table 4:** Predicted optimal colony size as the function of the consumption rate.

Optimization run:	5	6	7	8
Consumption rate:	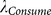 * = 0.01*⋅*min^−1^*	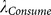 * = 0.02*⋅*min^−1^*	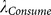 * = 0.04*⋅*min^−1^*	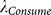 * = 0.08*⋅*min^−1^*
Max. payoff found at:	n_Colony_^*^ *** = 360*** ** ***ants***	n_Colony_^*^ *** = 191 ants***	n_Colony_^*^ *** = 99 ants***	n_Colony_^*^ *** = 51 ants***
The final payoff predicted:	*H(t_max_)* *** = 3495.9*** transported corpses per worker.	*H(t_max_)* *** = 6254.1*** transported corpses per worker.	*H(t_max_)* *** = 11013.1*** transported corpses per worker.	*H(t_max_)* *** = 18892.9*** transported corpses per worker.

The variable *n_Colony_* ^*^ indicates the optimal value of *n_Colony_* for a given value of 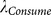, which was identified by a Powell optimization algorithm [51] for yielding the highest predicted value of *H(t_max_).*

### 5. Further discussion and conclusions

The collective foraging of *Ectatomma ruidum* is a clear example of a system that shows a distributed form of self-regulation. It is the availability (density) of prey and corpses that regulates the foraging activities for these very same items. The fluxes of prey and corpses also regulate the task partitioning associated with hunting behavior of these ants [60], [27]. This is very different from other mechanisms of self-regulation of collective foraging in social insects, such as the pheromone trails used in food site selection in many ant species [61], [62] or the waggle dances used for nectar source selection in honeybees [4], [6]. The dynamics, diversity, efficiency and robustness of such collective foraging decisions have been studied by mathematical modeling for ants [63], [64] and honeybees [45], [65], [66], [67], [68], [69], [70]. In some studies, also the importance of the underlying social structure, age-structure and size of the colony was studied [69], [71]. In all of these natural systems of collective decision making and self-regulation, a separate communication channel (pheromones, dances) is used to regulate the behavior of foragers, what can make the system vulnerable to wrong or out-dated information. To prevent this, workers frequently re-evaluate the communicated information by costly and repeated re-visits of the food source.

*Ectatomma ruidum* is using a simpler, but still flexible, reliable and very robust way to regulate hunting behavior. The inflow of nutrients is regulating itself through task partitioning performed by workers which are involved in the collective hunting. No additional communication channel is required, thus also no synchronization between multiple communication channels and no re-evaluations are necessary. We call such systems, in which a material is regulating its own foraging to be a “common stomach” [29], [42], [31], [32], [72]. Sometimes such systems are also called “social crop”, for example in the context of trophallactic food exchange in honeybees [44], [43]. Such substance-driven self-regulation of division of labor and task-partitioning was studied in several mathematical models in honeybees [73], [74], [75] and in wasps [29], [42], [31], [32], [72], [76], leading also to bio-inspired applications of comparable self-regulation mechanisms [77], [78]. In honeybees, the model of Johnson [79], [80] investigated how random walks and task abandonment can regulate task allocation in a demand-driven way for honeybees, while the models of Tofts [81] and of Franks and Tofts [82] explore the interplay of (random) worker locomotion and task recruitment in ants.

Task allocation is not only studied in mathematical models, but also in bio-inspired robotic models. For example, a swarm of autonomous robots that randomly pick up non-moving items in the environment and that communicate indirectly through the resulting changes in the environmental availability of these “prey items” was demonstrated and analyzed by Labella et al. [83]. To our knowledge, this was the first demonstration of an engineered system that is self-regulating its own task-allocation and activity of workers (robots) through a common-stomach like system, similar to the system we analyzed here in *Ectatomma ruidum* collective foraging and transportation.

When we designed the model, we identified four principles as guidelines to design the model’s structure along. The first principle is about **robust processes**. Any flow of material, any process of conversion of material as well as any recruitment and abandonment process will run with a specific rate. This rate depends on the availability of material or workers at the source, on the transport or conversion speed and also on the already achieved saturation at the target. No matter to which values these rates are set, some boundaries in the predicted model’s behavior should be met: (1) It shall never happen that more material is converted or transported than is available at the source. (2) It shall never happen that more target material is produced than fits the target site or compartment. (3), Finally, material and workers shall never get lost throughout the process or enter it, except in a well defined (modeled) way through a sink or through a source. In short, this means that whatever the value of a rate is, the model shall never achieve negative stocks or other impossible states. For all rates and parameters this holds for our model specifically between the natural boundaries of the values ***0*** and ***1***, which mean “nothing is converted” and “everything is converted”. As long as our model is parameterized within these natural boundaries, all predicted dynamics obey the **principle of robust processes**, as was shown in extensive parameter analyses (Figs. 3–10, S1 Text, S2 Text), while it does not hold for the previously published model [38] of the foraging of *Ectatomma ruidum* (S2 Text).

The second principle emphasized the role of **shared substances** as a communication channel for the homeostatic regulation of the system. The local availability of the shared substances are expressed by the saturation level of three common stomachs 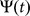, 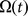, 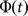, which in fact regulate all important conversion processes: prey to corpses, undecided ants to stingers and vice versa, undecided ants to transporters and vice versa. Transportation of corpses to the nest is also regulated in a similar way. All of these regulation processes are modeled on the assumption that either randomly moving ants meet prey or corpse items or they are based on defined time delays concerning the time how long workers stay in the task they are currently engaged in. Both principles can be easily described and modeled as localized individual events, without requiring any global communication and overview of the overall system, thus the principle of **localized interactions** is met throughout our model design. Finally, we required that the model’s design as well as its prediction respect the principle of **natural assumptions**. To adhere to this principle we checked all parameter values for their biological plausibility and reflected all known parameters (like dimensions of the foraging site or transportation rates) that we found in literature. Not a single parameter of our model was introduced just to fit or normalize a curve, all parameters are biologically plausible and well measurable in the real system.

Besides our 4 model design objectives, which we stated in detail in section called Model design principles, our model approach followed also the design principles suggested by Gilpin and Ayala [41]: (1) Simplicity: From all different possible model configuration, we have chosen a straight-forward model structure based on minimalistic mechanistic assumptions on the ants and the environment. We assumed no memory or any other form of higher cognitive functionality of the modeled ants as well as simple random-walk motion and mass action laws (2) Reality: We laid strong emphasize on assuming natural parameter values in the parametrization of our model. (3) Generality: Our extensive parameters sweeps and sensitivity analysis show that our model works with a wide range of parameter value combinations without predicting inplausible results. (4) Accuracy: Our predicted system behavior was extensively compared to empirical studies including some that were not used to construct or parametrize our model.

When we compared our model’s stability to the model of Theraulaz et al. [38], we found our model to be more robust. The authors of this previous model had a very specific goal in mind, namely to mimic the empirical experiments which they performed on colonies of *Ectatomma ruidum* and their model fulfilled this goal very well. Their model was not made to handle any sort of external perturbations, therefore we did not implement such perturbations in the sensitivity runs which we performed with the Theraulaz et al. [38]. The results show that even small deviations of their model’s parameters often cause unrealistic predictions. For example, many runs predict negative numbers for prey and corpse items (S2 Text). Our analyses indicate that more than 50% of all tested parameter combinations lead to unplausible negative values for important system variables (S2 Text). In this anaysis all sampled parameters were in the same plausible value range which we used in our model before (S2 Text). Our model did not show a single run exhibiting such an unplausible system behavior fulfilling our objective 1 (“principle of robust processes”) and objective 4 (“principle of natural assumptions”). This indicates that the structure of the Theraulaz et al. [38] model is simple enough to mimic their empirical results, but it seems to be too oversimplified for performing extensive (or exhaustive) evaluation of the hunting system of *Ectatomma ruidum.* Thus, in its published form, it does not look suitable for providing new predictions that are beyond the scope of their specific goal.

The way how our model is formulated offers several possible extensions of the model, which would allow to investigate the impact of the common stomach regulation on ecological relevant time scales and on intra-colonial population dynamics:

1. Brood production: In times when more corpses are delivered into the colony more brood can be produced by the workers and by the queen. In consequence, this higher number of brood consumes the collected corpses faster, what in turn potentially will lead to a self-regulation of brood and corpses in the nest.
2. Colony growth: Successfully raised brood will hatch into new workers and these workers will in turn affect brood production and collective hunting capabilities. However, colony growth is also affected by the average prey influx to the hunting zone, therefore an interesting interplay between new positive and the negative feedback loops can be expected for such a model extension.
3. Reproduction of prey: In our current model the influx of prey assumes only (linear) immigration of prey from other habitats, because there is no positive feedback loop of growth modeled for the prey population. Such an implementation of prey reproduction would also decrease the models sensitivity to *n_Colony_* and *A_Arena_*, as prey would “automatically” converge towards an equilibrium density of prey that is regulated by the ant colony as their major predator.

We have to point out however, that all of these extensions would require the model to operate on a different time scales, having 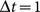 day and runing simulations for several weeks or months.

Our model is an example of a natural decentralized system that is able to self-regulate in a robust and flexible way. Despite these significant aspects, the mechanisms to establish such a system are rather simple, thus, they are assumed to be easily implementable also in distributed engineered systems, like swarm robotics (see for example [83], [77], [78]. Thus, understanding the functioning of such biological model systems does not only enhance biological knowledge by allowing novel interpretations in ecology and evolution of social insects, it may also enhance the development of bio-inspired engineered systems, like it was shown for optimization algorithms [84], [85], communication networks [86] and collective robotics [87], [88].

## Supporting Information

S1 Figure **Results of parameter sweeps of all model parameters.** The graphs display the final equilibrium values at *t_max_* **= **500 min for the three task groups (*T, U, S*). In each graph, only the indicated parameter was varied, all other parameter values are kept on their default values (Table 1). Solid line: *S(t_max_),* dashed line: *U(t_max_);* dotted line: *T(t_max_)*.
(EPS)

S2 Figure **Results of parameter sweeps of all constant model parameters.** This graph displays the final equilibrium values at t_max_ = 500 min for the three food-associated system variables (*P*, *C*, *N*). In each graph, only the indicated parameter was varied, all other parameter values are kept on their default values (Table 1). Solid line: *P(t_max_)*, dashed line: *C(t_max_)*; dotted line: *N(t_max_)*.
(EPS)

S3 Figure
**Sensitivity analysis of our model of the collective foraging of** ***Ectatomma ruidum*** **.** The same perturbations were performed as they shown in Fig. 10. In addition to that we varied our model’s key parameters (all *K*, 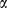, 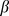 and 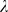 values) in a random uniform manner within the range of ±50% around their default values (Table 1) using a Latin Hypercube sampling method. The black region contains the predictions of 33% of all 1000 simulation runs. The dark gray region contains 66%, the medium gray region contains 95% and the light gray regions contains all predictions.(TIF)

S4 Figure **Sensitivity analysis of the original model of Theraulaz et al.** [38] **on the collective foraging of** ***Ectatomma ruidum*** **.** We varied the model’s key parameters (recruitment rates, abandonment rates, stinging success rate and transportation rates) in a random uniform manner within the range of ±50% around their default values using a Latin Hypercube sampling method. The black region contains the predictions of 33% of all 1000 simulation runs. The dark gray region contains 66%, the medium gray region contains 95% and the light gray regions contains all predictions.
(TIF)

S1 Text **Single parameter sweeps.**
(DOCX)

S2 Text **Sensitivity analysis.**
(DOCX)
