# Supplementary figures and images for: Sting, Carry and Stock: How Corpse Availability Can Regulate De-Centralized Task Allocation in a Ponerine Ant Colony
Source: PLoS One. 2014 Dec 10;9(12):e114611. doi: 10.1371/journal.pone.0114611 (PMC4262436; doi:10.1371/journal.pone.0114611)

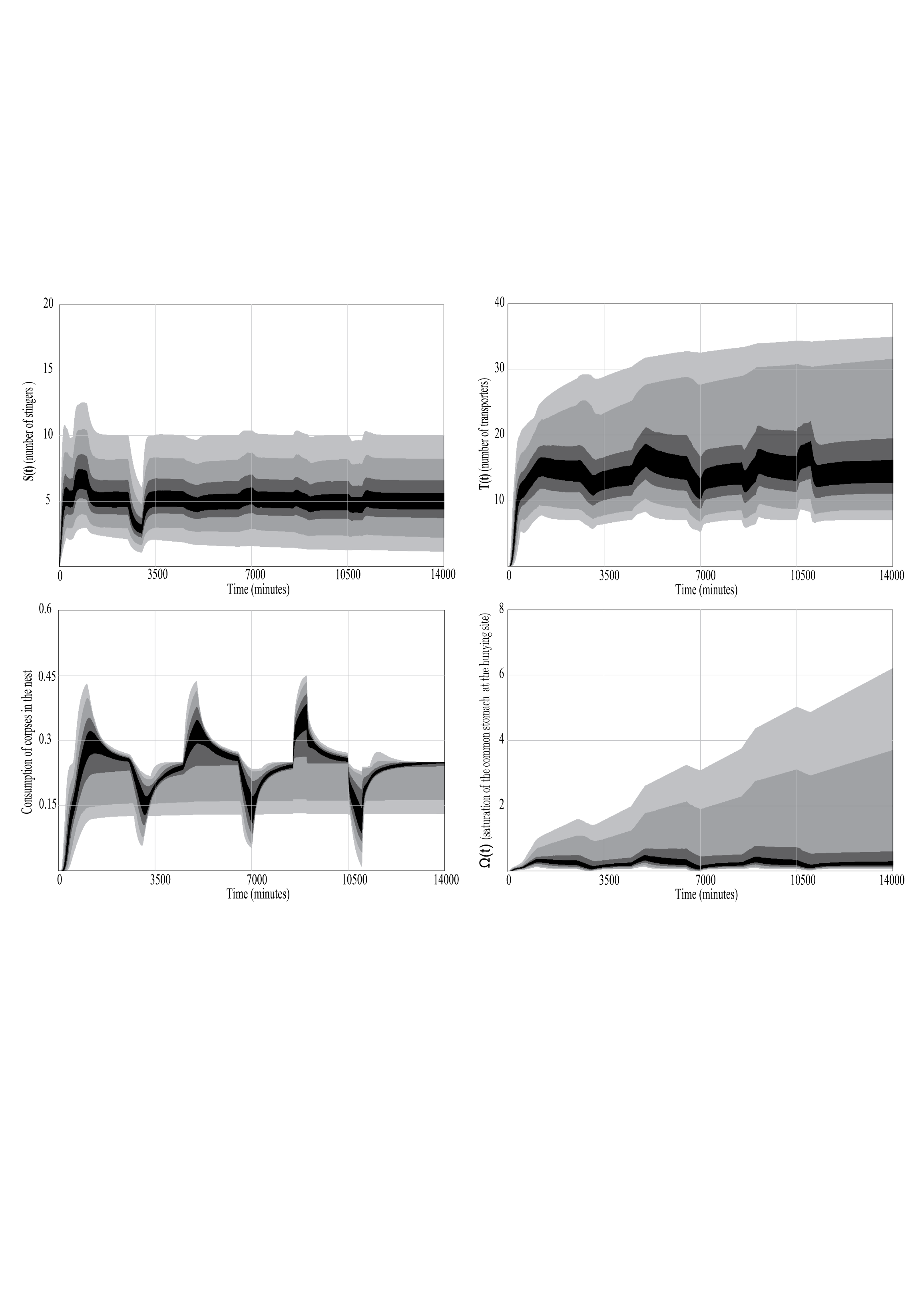

Supplement: S3 Figure — Sensitivity analysis of our model of the collective foraging of Ectatomma ruidum . The same perturbations were performed as they shown in Fig. 10. In addition to that we varied our model’s key parameters (all K, , and values) in a random uniform manner within the range of ±50% around their default values (Table 1) using a Latin Hypercube sampling method. The black region contains the predictions of 33% of all 1000 simulation runs. The dark gray region contains 66%, the medium gray region contains 95% and the light gray regions contains all predictions. (TIF) [file pone.0114611.s003.tif]

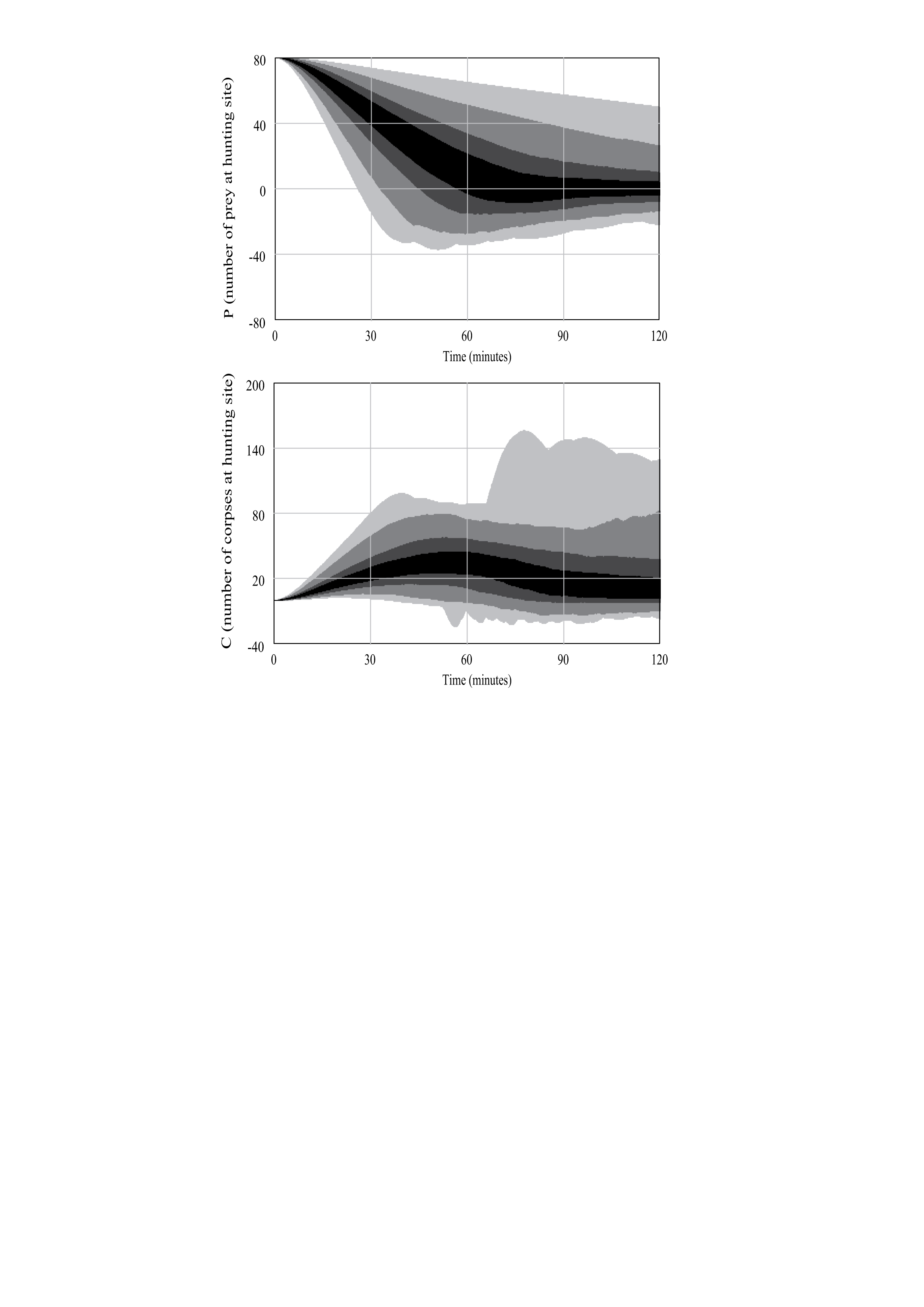

Supplement: S4 Figure — Sensitivity analysis of the original model of Theraulaz et al. [38] on the collective foraging of Ectatomma ruidum . We varied the model’s key parameters (recruitment rates, abandonment rates, stinging success rate and transportation rates) in a random uniform manner within the range of ±50% around their default values using a Latin Hypercube sampling method. The black region contains the predictions of 33% of all 1000 simulation runs. The dark gray region contains 66%, the medium gray region contains 95% and the light gray regions contains all predictions. (TIF) [file pone.0114611.s004.tif]
