# Supplementary material for: Sting, Carry and Stock: How Corpse Availability Can Regulate De-Centralized Task Allocation in a Ponerine Ant Colony
Source: PLoS One. 2014 Dec 10;9(12):e114611. doi: 10.1371/journal.pone.0114611 (PMC4262436; doi:10.1371/journal.pone.0114611)
Supplement: S1 Text — Single parameter sweeps. (DOCX) [file pone.0114611.s005.docx]

Text S1. **Single parameter sweeps.**

In this supplement we describe how our model behaves when a single parameter is adjusted (parameter sweeps) (Fig. S1, Fig. S2) We report how the equilibrium levels that were reached at *t_max_ = 500* minutes correlate with the setting of the focal parameters (Table 2). For the sake of a concise article, we go into detailed discussion where the results show either complex responses (correlations do not hold for the whole parameter space) or where we found counter-intuitive results.

$\alpha_{Stingers}$: An increase of the recruitment rate of stingers ($\alpha_{Stingers}$) leads, as expected, to a significantly higher number of stingers (*S*) and to a significantly lower number of undecided hunters (*U*). However, the number of transporters (*T*) is predicted to be almost unaffected. These results indicate that the number of transporters is strongly regulated towards an equilibrium which is mainly governed by the availability of prey (influx) which in turn determines the corpse influx. An increase of the recruitment rate of stingers prominently reduces the number of available prey (*P*), especially in the range of *0* ***≤*** $\alpha_{Stingers}$ ***≤*** *0.05*) while it affects the number of corpses at the hunting site (*C*) and in the nest (*N*) only weakly (Table 2). A special case is $\alpha_{Stingers}=0$: As there are no stingers recruited, no corpses are accumulated at the hunting site, thus also no transporters are needed and no corpses appear in the nest. Any positive value of $\alpha_{Stingers}$results in an equilibrium of our system variables (*S*, *T*, *U*, *P*, *C*, *N*), indicating that these equilibria have a large basin of attraction and that they are very robust. Strong correlations are expressed in the range of *0* ***≤*** $\alpha_{Stingers}$ ***≤*** *0.2* for the worker cohorts, while strong correlations are expressed in the range of *0* ***≤*** $\alpha_{Stingers}$ ***≤*** *0.1* for the food stocks.

$\beta_{Stingers}$: An increase of the abandonment rate of stingers ($\beta_{Stingers}$) should result in similar results as an increase of the respective recruitment rate $\alpha_{Stingers}$. This was observed for all our focal system variables except for the number of transporters (*T*), which decreases with increasing values of $\beta_{Stingers}$. This is an expected result for an increased abandonment rate. However, it is noteworthy that a decreased recruitment rate ($\alpha_{Stingers}$) did not have such an effect because the modeled common stomach regulation still leads to sufficient recruitment of transporters in such situations.

$\alpha_{Transporters}$: An increase of the recruitment rate of transporters ($\alpha_{Transporters}$) leads to a very slight decrease of the number of stingers (*S*), to a very strong increase of the number of transporters (*T*) and to a strong decrease of the number of undecided ants (*U*). In consequence, the number of prey items at the hunting site (*P*) increases, the number of corpses at the hunting site (*C*) strongly decreases and the number of corpses in the nest (*N*) stays almost unaffected. A special case is $\alpha_{Transporters}=0$: As there are no transporters recruited, no corpses are accumulated in the nest. As soon as $\alpha_{Transporters}$ is on a value being only slightly above *0*, the previously described equilibria emerge, indicating that these equilibria have a large basin of attraction and are very robust. Strong correlations are expressed in the range of *0* ***≤*** $\alpha_{Transporters}$ ***≤*** *0.3* for the worker cohorts, while strong correlations are expressed in the range of *0* ***≤*** $\alpha_{Transporters}$ ***≤*** *0.1* for the food stocks.

$\beta_{Transporters}$: An increase of the abandonment rate of transporters ($\beta_{Transporters}$) is expected to result in similar results as an increase of the respective recruitment rate $\alpha_{Transporters}$. In fact, this was observed for all system variables except for the number of transporters.

*n_Colony_*: An increase of the colony size (*n_Colony_*) leads to a weak increase of the number of stingers (***S***), to a strong increase of the number of transporters (*T*) and to a very strong increase of the number of undecided ants (*U*). In consequence, the number of prey items at the hunting site (*P*) strongly decreases. The number of corpses at the hunting site (*C*) shows a complex pattern: For the range of *1 ≤ n_Colony_ ≤ 125* the predicted value of *C* increases while for higher values of *n_Colony_* the predicted value of *C* decreases again. In contrast, the number of corpses accumulated in the nest (*N*) steadily increases with colony size. The correlations between worker cohorts and colony size are close to linear, thus showing no saturation effects in the analyzed range. In contrast to that, the correlation between prey items (*P*) and colony size is strongly non-linear, especially for values of *1 ≤ n_Colony_ ≤ 125*. From these parameter sweeps we conclude that at the given prey influx and consumption rate there is a colony size (here: *n_Colony_ = 125 ants)*, where the colony works with maximum efficiency. A further increase of the colony size leads to an “overfilling” of the colony depending on the chosen value of *K_Nest_*.

*K_Prey_*: An increase of the saturation capacity of prey at the hunting site (*K_Prey_*), which describes a threshold level for maximum recruitment of stingers through prey density, leads to a lower number of stingers (*S*), to a lower number of transporters (*T*) and to a higher number of undecided workers (*U*). In consequence, the number of living prey animals at the hunting site (*P*) increases and the number of corpses at the hunting site (*C*) and in the nest (*N*) decreases. Strong correlations are expressed in the range of *15 ≤ K_Prey_ ≤ 80* for the worker cohorts, while strong correlations are expressed in the range of *0 ≤ K_Prey_ ≤ 40* for the food stocks.

*K_Corpses_*: An increase of the saturation capacity of corpses at the hunting site (*K_Corpses_*), which describes a threshold level for maximum transporting through corpse density at the hunting site, leads to a very weak decrease of the number of stingers (*S*), to a decrease of the number of transporters (*T*) and to an increase of the number of undecided ants (*U*). In consequence, the number of prey items at the hunting site (*P*) decreases slightly, the number of corpses at the hunting site (*C*) increases significantly while the number of corpses in the nest (*N*) decreases. Strong correlations are expressed in the range of *15 ≤ K_Corpses_ ≤ 50* for the worker cohorts, while strong correlations are expressed in the range of *0 ≤ K_Corpses_ ≤ 15* for the food stocks.

*K_Nest_*: An increase of the saturation capacity of corpses in the nest (*K_Nest_*), which describes a maximum saturation of the nest with corpses (*K_Nest_*) leads to a very weak decrease of the number of stingers (*S*), to a significant increase of the number of transporters (*T*) and to a decrease of the number of undecided ants (*U*). In consequence, the number of prey items at the hunting site (*P*) increases slightly, the number of corpses at the hunting site (*C*) decreases significantly while the number of corpses in the nest (*N*) increases. Strong correlations are expressed in the range of *0 ≤ K_Nest_ ≤ 0.2* for the worker cohorts while strong correlations are expressed in the range of *0 ≤ K_Nest_ ≤ 0.1* for the food stocks.

$\xi_{\Pr ey}$: An increase of the influx of prey to the hunting site ($\xi_{\Pr ey}$) leads to a higher number of stingers (*S*), to a higher number of transporters (*T*) and to a lower number of undecided ants (*U*). In consequence the number of prey items at the hunting site (*P*) increases due to the higher influx, indicating that the increased number of stingers cannot compensate for all the increased influx, because more stingers produce more corpses at the hunting site (*C*) which require also transporters to emerge in higher numbers. At a given colony size and at a certain point of influx ($\xi_{\Pr ey}$ > 0.3) the number of workers is not high enough to accomplish both tasks sufficiently well therefore the number of corpses at the hunting site (*C*) starts to accumulate as the influx of prey is increased, while the number of corpses accumulated in the nest (*N*) increases only slightly.

$\xi_{Corpses}$: An increase of the influx of corpses to the hunting site ($\xi_{Corpses}$) leads to almost no changes in the number of stingers (*S*), to a higher number of transporters (*T*) and to a lower number of undecided ants (*U*). In consequence the number of prey items at the hunting site (*P*) is almost unaffected by increases of *ξ_Corpses_*, as is also the number of corpses in the nest (*N*). However, the number of corpses at the hunting site (*C*) is strongly affected by additional corpse influx, growing even super-linear, at least for corpse influxes below $\xi_{Corpses}$ *< 0.4*.

$\xi_{Nest}$: An increase of the influx of corpses into the nest ($\xi_{Nest}$) leads to almost no changes in the number of stingers (*S*), to a very small decrease in the number of transporters (*T*) and to a very small increase in number of undecided ants (*U*). In consequence the number of prey items (*P*) is almost unaffected by an increase of $\xi_{Nest}$, while the number of corpses at the hunting site (*C*) as well as the number of corpses in the nest (*N*) linearly increases with increasing values of $\xi_{Nest}$.

$\lambda_{Kill}$: An increase of the maximum rate of stinging ($\lambda_{Kill}$) leads to a strong decrease of the number of stingers (*S*) and to a strong increase of the number of transporters (*T*). This is plausible as higher killing efficiency reduces the required number of stingers while it increases the demand for the transportation task in the colony. The number of undecided ants shows a short (negative) peak at the point where *S* and *T* intersect, indicating that at this value of $\lambda_{Kill}$ the highest fraction of workers is predicted. In consequence the number of prey items (*P*) decreases strongly with increasing stinging efficiency, while the corpses at the hunting site and in the nest (*C*,*N*) increase with increasing values of $\lambda_{Kill}$. A special case is $\lambda_{Kill}=0$, when there can be no prey items killed and thus no corpses are produced. This leads to predicted values of (*T*, *C*, *N) = (0*, *0*, *0)*.

$\lambda_{Transport}$: An increase of the maximum rate of transportation ($\lambda_{Transport}$) leads to a weak increase in the number of stingers (*S*) and to a strong decrease in the number of recruited transporters (*T*). This is plausible because higher transportation efficiency reduces the required number of transporters and this in turn allows more ants to be recruited for the stinging task. The number of undecided ants shows a strong increase with increasing values of $\lambda_{Transport}$ indicating that the transportation task binds most of the workforce in the system. In consequence the number of prey items (*P*) decreases weakly with increasing transporting efficiency, the number of corpses at the hunting site (*C*) decreases strongly while the number of corpses in the nest (*N*) increases significantly with increasing values of $\lambda_{Transport}$. A special case is $\lambda_{Transport}=0$, when there can be no corpses transported to the nest (*N(t_max_) = 0*).

$\lambda_{Consume}$: An increase of the consumption rate of corpses in the nest ($\lambda_{Consume}$) results in an almost unaffected number of stingers (*S*), in a strong increase in the number of transporters (*T*) and in a strongly decreased number of undecided ants (*U*). This is plausible as higher consumption in the nest prevents the nest from saturating with corpses and thus allows higher transportation flux of corpses into the nest. In consequence the number of prey items (*P*) decreases only weakly with increasing consumption rate. The number of corpses at the hunting site (*C*) decreases strongly while the number of corpses in the nest (*N*) decreases even stronger with increasing values of $\lambda_{Consume}$.

$A_{Arena}$: An increase of the size of the hunting site ($A_{Arena}$) without changing the prey influx results in weak decrease of the number of stingers (*S*), in a significant decrease in the number of transporters (*T*) as well as in a significant increase in the number of undecided ants (*U*). This is plausible because any increase in the size of the hunting area decreases the spatial density of prey, which was identified being a key factor for the hunting success. In consequence the number of living prey items at the hunting site (*P*) increases strongly until the equilibrium prey density is reached again for the larger arena size. The number of corpses at the hunting site (*C*) shows a rather complex relationship to different levels of size of the hunting site: For small arenas (*10cm^2^ ≤* $A_{Arena}$ *≤ 100 cm^2^*) the number of accumulated corpses at the hunting site increases, while for larger arena sizes ($A_{Arena}$ *> 100 cm^2^*), the final values of this system variable *C* decrease again. The number of corpses accumulating in the nest (*N*) decreases for the parameter range (*10cm^2^ ≤* $A_{Arena}$ *≤ 100 cm^2^*), with larger arena sizes it shows an equilibrium of *N=0* prey items, indicating that in case of low prey density the colony would starve.
