# Supplementary material for: Sting, Carry and Stock: How Corpse Availability Can Regulate De-Centralized Task Allocation in a Ponerine Ant Colony
Source: PLoS One. 2014 Dec 10;9(12):e114611. doi: 10.1371/journal.pone.0114611 (PMC4262436; doi:10.1371/journal.pone.0114611)
Supplement: S2 Text — Sensitivity analysis. (DOCX) [file pone.0114611.s006.docx]

**Sensitivity analysis**

Many parameters we used in our model are based on experiments and observations on real colonies (Table 1). However several other parameters are assumptions or are derived from empirical data indirectly for example by non-linear regression analysis. In field and lab experiments, recruitment and abandonment rates, carrying capacities or killing rates are usually not easily measurable in the real system and therefore such rates can only be estimated up to a certain degree of accuracy. This can cause a significant level of uncertainty concerning the natural values of these parameters. To deal with this issue explicitly, we performed a focused set of sensitivity analyses with our model. This analysis is conducted along two design objectives: (1) The analysis investigates those parameters that are hard to measure in the real system and which are important components of our model (all *K*, $\alpha$, $\beta$and $\lambda$ values) and (2) it measures the impact of these parameters on the system behavior only in a defined neighborhood around an estimated mean value. Thus, our focal parameters were varied uniformly within the range of ±50% around their default values (Table 1). Using a sensitivity analysis tool, which is built into the software Vensim [51], we performed 1000 simulation runs, in which the initial values of the investigated parameters were sampled and combined randomly (random uniform distribution) with the Latin-Hypercube sampling method. In these sensitivity runs we also included the same external perturbations we carried out previously (see section called ‘Perturbation experiments’, Fig. 10), i.e., we perturbed the common stomachs with a series of pulsed additional flows (in or out) having an amplitude of ±0.2 prey/min for a duration of 500 min in order to carry out strong testing scenarios.

Our analysis shows that the model system is behaving in a large variety of ways under the regime of the sampled parameter combinations (Fig. S3). However, the observed variance in the system behavior is smaller than the induced variance in the parameter sampling. Most perturbations are clearly detectable in the system’s reactions (Fig. S3) and the resulting system behaviors of 66% of all runs (ribbons in black and darkest gray in Fig. S3) are falling within a narrow gray ribbon around the central base line. These results indicate that the model is not sensitive to our random changes of the model’s parameters. Looking at all runs as a aggregated result, it is clear that none of the random combinations of parameters led to unrealistic, thus unnatural, system behaviors. The numbers of predicted stingers and transporters as well as the consumption of corpses show always a positive value and none of them leaves the range of biological plausible values. The dynamics of the saturation of the common stomach formed by corpses at the hunting site **(**$\Omega(t)$) show self-regulation around an equilibrium level in 95% of all sampled parameters and linear accumulation in 5 percent of the simulation runs that had the most extreme parameter combinations (Fig. S3, lower right figure). In these extreme parameters settings, more prey entered the system that could be effectively transported to the nest, thus the corpses were predicted to accumulate. While these are extreme predictions, they still can represent natural results in extreme situations and they are obeying all necessary conservation laws.

In addition to the sensitivity analysis of our own model, we also re-implemented the model described by Theraulaz et al. [38] in Vensim [51] and performed a similar sensitivity analysis with it. From this analysis (Fig. S4) we conclude that our model shows significantly more structural stability than the model of Theraulaz et al. [38].
